# Relationship between Biochemical Pathways and Non-Coding RNAs Involved in the Progression of Diabetic Retinopathy

**DOI:** 10.3390/jcm13010292

**Published:** 2024-01-04

**Authors:** Małgorzata Mrowicka, Jerzy Mrowicki, Ireneusz Majsterek

**Affiliations:** Department of Clinical Chemistry and Biochemistry, Medical University of Lodz, Mazowiecka 5, 92-215 Lodz, Poland; jerzy.mrowicki@umed.lodz.pl (J.M.); ireneusz.majsterek@umed.lodz.pl (I.M.)

**Keywords:** diabetic retinopathy, biochemical pathways, oxidative stress, ncRNAs, biomarkers

## Abstract

Diabetic retinopathy (DR) is a progressive blinding disease, which affects the vision and quality of life of patients, and it severely impacts the society. This complication, caused by abnormal glucose metabolism, leads to structural, functional, molecular, and biochemical abnormalities in the retina. Oxidative stress and inflammation also play pivotal roles in the pathogenic process of DR, leading to mitochondrial damage and a decrease in mitochondrial function. DR causes retinal degeneration in glial and neural cells, while the disappearance of pericytes in retinal blood vessels leads to alterations in vascular regulation and stability. Clinical changes include dilatation and blood flow changes in response to the decrease in retinal perfusion in retinal blood vessels, leading to vascular leakage, neovascularization, and neurodegeneration. The loss of vascular cells in the retina results in capillary occlusion and ischemia. Thus, DR is a highly complex disease with various biological factors, which contribute to its pathogenesis. The interplay between biochemical pathways and non-coding RNAs (ncRNAs) is essential for understanding the development and progression of DR. Abnormal expression of ncRNAs has been confirmed to promote the development of DR, suggesting that ncRNAs such as miRNAs, lncRNAs, and circRNAs have potential as diagnostic biomarkers and theranostic targets in DR. This review provides an overview of the interactions between abnormal biochemical pathways and dysregulated expression of ncRNAs under the influence of hyperglycemic environment in DR.

## 1. Introduction

In recent years, a systematic increase in the incidence of diabetes mellitus (DM) has been observed, and this disease is considered a global epidemic. According to estimates, in 2021, around 537 million people (aged 18–99) in the world suffered from DM. Almost half (49.7%) of all diabetic patients are still undiagnosed. It is projected that by 2045, the number of patients with DM will have increased to approx. 783 million [1].

DM is accompanied by many complications, which are the main cause of chronic diseases, including eye diseases. DM can lead to many serious ocular complications, including diabetic retinopathy (DR), neovascular glaucoma (NVG), cataracts, and ocular surface changes. Due to poor glycemic control and increasing insulin resistance, diabetic retinopathy, being one of the main microvascular complications, is becoming the leading cause of blindness in the population of professionals in urban areas [2]. The longer the duration of diabetes, the more often DR occurs. Epidemiological studies estimate that 20 years after the diagnosis of the disease, almost all patients with type 1 DM and approximately 80% of patients with type 2 DM have demonstrated changes related to diabetic retinopathy [3]. Patients diagnosed with diabetes at a young age are at greater risk of retinopathy and its progression. Patients with type 2 diabetes develop retinopathy early, i.e., within 3 years following its diagnosis, 12% of people develop changes in the eye fundus. Due to the overall rapid increase in the incidence of diabetes, by the year 2045, the global number of adults affected by DR, vision-threatening DR, and diabetic macular edema (DME) is projected to increase to approximately 161 million, 45 million, and 29 million, respectively [4].

Diabetic retinopathy (DR) is a complex microvascular disease characterized by various clinical manifestations and biochemical pathways, which contribute to its pathophysiology. The exact mechanism responsible for the development and progression of diabetic retinopathy is not fully understood, but extensive research has shed light on several key molecular processes involved. Moreover, DR has long been considered a microvascular complication of diabetes; however, growing evidence suggests that neurodegeneration is an early event in its pathogenesis [5,6]. DR is a common and debilitating microvascular complication of both type 1 and type 2 diabetes. Abnormalities in the retinal function can be detected in patients without any evidence of microvascular abnormalities, and the American Diabetes Association (ADA) has defined DR as a highly specific neurovascular complication. Thus, in view of current knowledge on microangiopathic changes in the eye fundus, diabetic retinopathy is perceived as a neurodegenerative disease [7,8]. Abnormalities in the structure and function of the retinal blood vessels in diabetic patients are manifested by deterioration of visual acuity, reduced contrast sensitivity, delayed dark adaptation, and poorer color vision [9].

### 1.1. Factors, Which Increase Development of Diabetic Retinopathy

The development and progression of changes in diabetic retinopathy are associated with many factors. In the first place, the duration of the disease and the age at diagnosis of diabetes should be mentioned, as they are considered the main factors in the development of diabetic retinopathy. Furthermore, most people diagnosed with diabetes never undergo an eye check-up until required. A very complex relationship exists between an individual’s socioeconomic status and the disease [10]. Fluctuations in blood glucose levels, which have a negative impact on the state of retinal microcirculation and may lead to rapid disease progression, are the second risk factor [11]. In addition, a high level of glycosylated hemoglobin (HbA1c) may indicate the progression of changes in the eye fundus [12]. Other factors, which may contribute to the development of retinopathy, are higher systolic or diastolic blood pressure, hypertension [13], obesity [14], hyperlipidemia [15,16], anemia [17], pregnancy [18], puberty [19], cataract surgery [20], nephropathy [21]. The waist-to-hip ratio (WHR), an indicator of central obesity, has been found to be associated with insulin resistance and serves as an independent risk factor for retinopathy in diabetic patients. Furthermore, a combination of WHR, Hb A1c level, and hypertension has been identified as a risk factor for retinopathy [22]. DR and nephropathy have been shown to coexist in both type 1 and type 2 diabetes. DR may be the most common microcirculatory problem in diabetes, even more common than nephropathy [13].

Elevated blood pressure (BP) is also a recognized risk factor for DR progression in pregnancy (i.e., systolic BP > 115 mmHg in pregnant women in comparison to BP < 105 mmHg in non-pregnant women) [23].

In addition, studies have shown a significant association with sleep apnea [24], post-translational amendments of histones within chromatins [25], DNA methylation [26], and non-coding RNAs, such as miRNAs [27,28,29] and lncRNAs [30,31]. They all affect many biochemical pathways.

### 1.2. Classification of Diabetic Retinopathy

Diabetic retinopathy is a microangiopathy affecting particularly the small vessels of the retina. In the course of diabetic retinopathy, symptoms of obstruction and leakage of capillaries predominate. Structural and functional changes occurring in individual layers of the retina in diabetic patients appear before clinical manifestations of DR. Reduced retinal blood flow is observed in the early stages of the disease. Inflammatory changes, leukostasis, vascular occlusion, and neurodegenerative changes lead to hypoxia and further disease progression. As a result of remodeling of vascular walls and changes in blood rheology, perfusion is reduced in retinal ischemia [32].

DR is usually asymptomatic at the onset. Early clinical features of DR are visible during an ophthalmoscopic examination at the fundus. Diabetic retinopathy can be divided into non-proliferative (simple) retinopathy (NPDR), affecting the retina, and proliferative retinopathy (PDR), characterized by a loss of vision.

NPDR manifests by small hemorrhages in the middle layers of the retina, microaneurysms resulting from protrusion of a weakened retinal capillary wall, hard exudates formed by lipid material accumulating in the outer plexiform layer, areas of accumulation and stagnation of axoplasms of nerve fibers, lack of blood flow, and intraretinal microvascular abnormalities (IRMA). Retinal edema may result from microcirculatory exudate and is an indicator of impaired blood–retina barrier [33].

Proliferative diabetic retinopathy is associated with the proliferation of pathological vessels/neovascularization within the retina and/or optic disc. PDR may lead to preretinal hemorrhage and/or vitreous hemorrhage. New vessels traverse to the surface of the retina and into the vitreous body through cavities in its inner layers. The vitreous body shrinks, which additionally causes hemorrhages, thereby contributing to development of vascular-fibrous proliferation. As a consequence, part of the neurosensory retina becomes detached from its pigment epithelium. Without medical intervention, vision loss will occur because neurodegeneration will contribute to neuroapoptosis, which in turn will lead to death of retinal ganglion cells, glial alteration, and abnormalities in the retinal pigment layer. Furthermore, neovascularization may develop in the anterior segment of the eyeball, on the iris, as a result of severe changes in the eye fundus and intensive production of vascular endothelial growth factor (VEGF) [6].

### 1.3. Involvement of Biochemical Changes in Progression of DR

#### 1.3.1. Hyperglycemia and Hypoxia

**Figure 1:**
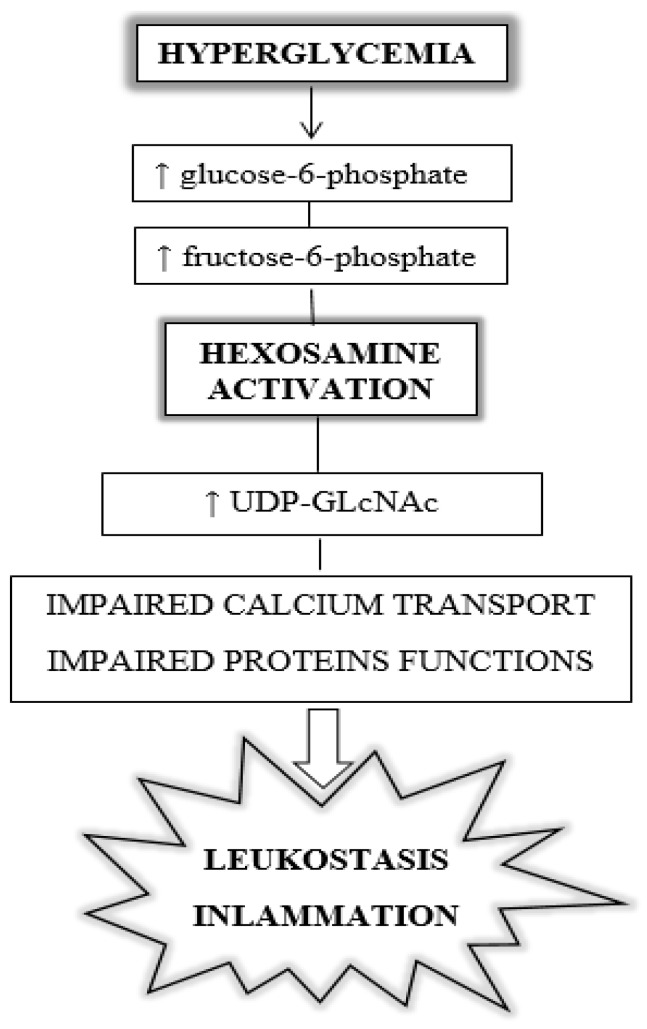
Diabetes-induced hexosamine pathway, leading to leukostasis and inflammation. UDP-GLcNAc: uridine diphosphate N-acetylglucosamine.

High glucose alters several metabolic pathways. Some of the major pathways implicated in the development of retinopathy include increased activity of the diacylglycerol (DAG) pathway and protein kinase C (PKC), accumulation of advanced glycation end products (AGEs), activation of the hexosamine pathway, overproduction of growth factors (vascular endothelial growth factor (VEGF) and insulin-like growth factor (IGF-1)), activation of the renin–angiotensin–aldosterone system (RAAS), accumulation of inflammatory mediators and sub-clinical inflammation with leukostasis, activation of the polyol pathway, and oxidative stress [34]. The induction of the hexosamine pathway by hyperglycemia leading to leukostasis and inflammation is shown in Figure 1.

At the beginning of the pathomechanism, hyperglycemia promotes microvascular degeneration and blood–retina barrier (BRB) breakdown. Endothelial cells of the retinal vessels constitute a tight barrier for blood components. Hyperglycemia leads to a series of changes in endothelial cells and their supporting cells, pericytes, which results in their dysfunction and death. Apoptosis of pericytes and endothelial cells, capillary occlusion, and increased vascular permeability also occur in the early non-proliferative phases of DR. Any changes causing, e.g., a formation of microaneurysms, reduce the efficiency of the supply of oxygen and nutrients to retinal cells. As a result of oxygen deficiency, deepening hypoxia stimulates the production of growth factors responsible for the formation of new blood vessels, i.e., angiogenesis [35,36].

#### 1.3.2. Disorders of the Polyol Pathway

**Figure 2:**
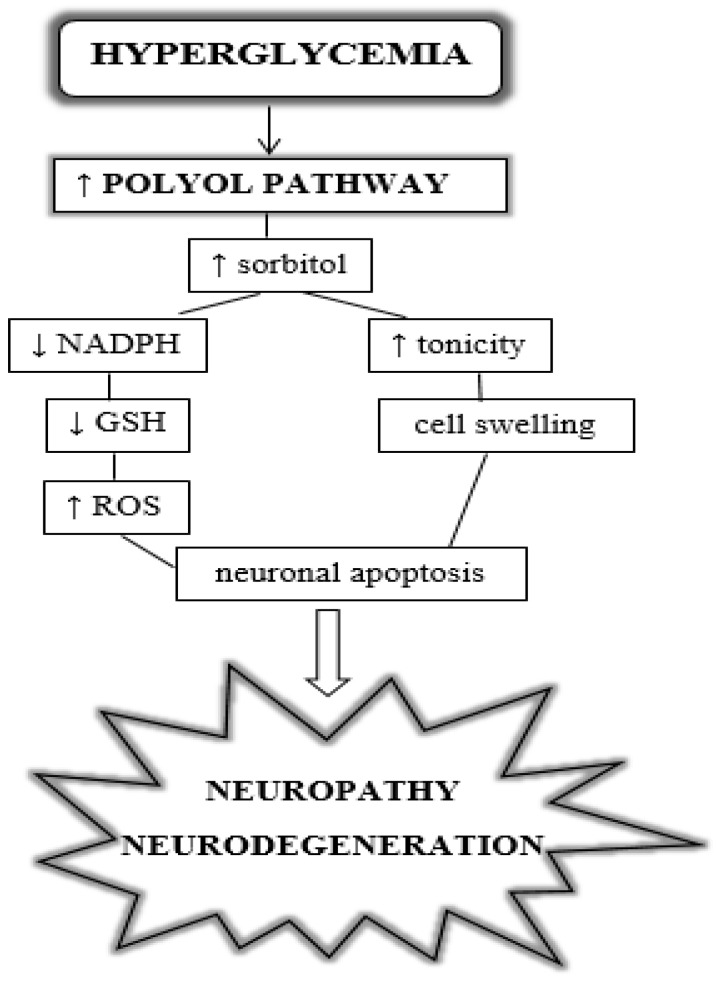
Diabetes-induced polyol pathway, leading to retinal neuropathy and neurodegeneration. NADPH: nicotinamide adenine dinucleotide phosphate; GSH: glutathione; ROS: reactive oxygen species.

Hyperglycemia also causes increased conversion of glucose to sorbitol and fructose via the polyol pathway and overexpression of enzymes, which are part of this pathway: aldose reductase (AR) and sorbitol dehydrogenase (SDH). These processes occur in tissues where glucose transport is independent of insulin (retina, eye lens, kidneys, peripheral nerves). The polyol pathway is activated in endothelial cells, pericytes, and Müller cells to produce macular edema and subsequent retinal ischemia. The concentration of sorbitol, fructose, and galactitol increases, which poorly penetrates cell membranes and results in accumulation, which leads to swelling and tissue damage (see Figure 2). Increased oxidation of nicotinamide adenine dinucleotide phosphate (NADPH) to NADP^+^ and reduction of NAD^+^ to NADH leads to tissue hypoxia [37]. Sorbitol, impermeable to cellular membranes, accumulates within the cell. This phenomenon is followed by the slow metabolism of sorbitol to fructose. Fructose produced by the polyol pathway can be phosphorylated to fructose-3-phosphate, which in turn can be degraded to 3-deoxyglucosone. These two are strong glycating agents and can contribute to the production of AGEs [38,39,40]. Studies have shown that increased AR activity is localized in, among others, pericytes, retinal endothelial cells, ganglion cells, Müller cells, retinal pigment epithelial cells, and neurons. AR hyperactivity is involved in the destruction of retinal cells [41]. Furthermore, the aldose reductase gene is characterized by the largest number of polymorphisms associated with diabetic retinopathy [42].

#### 1.3.3. Diacylglycerol and Kinase Pathway Protein C

**Figure 3:**
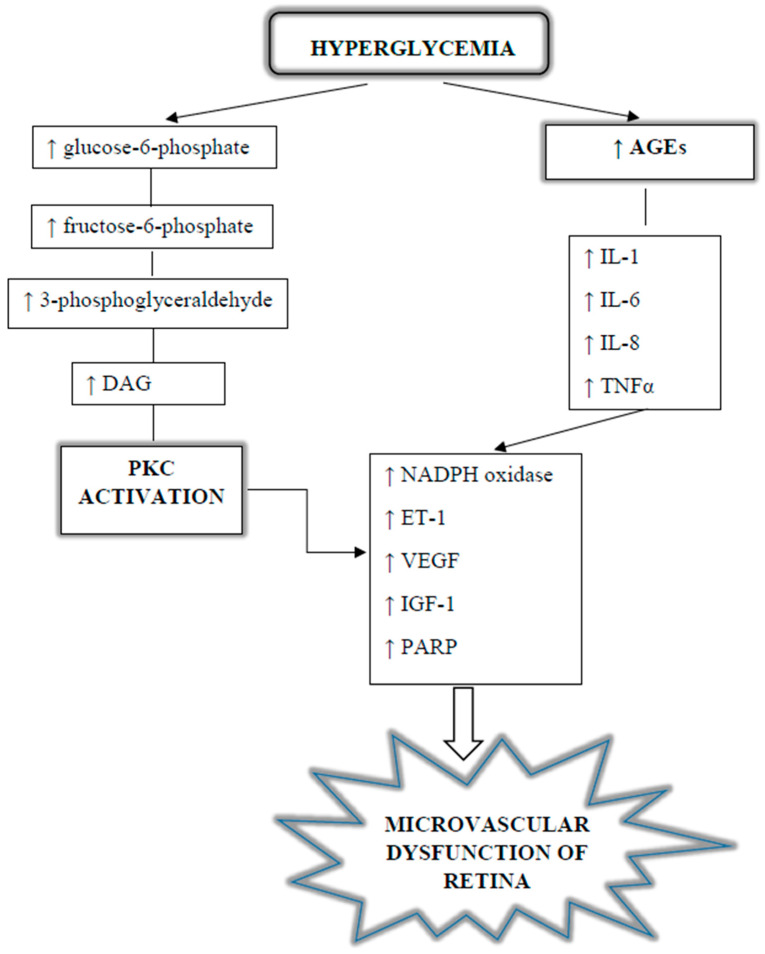
Diabetes-induced protein kinase C pathway (PKC) and AGEs formation, leading to microvascular dysfunction of retina. AGEs: advanced glycation end products; DAG: diglyceride; ET-1: endothelin-1; IGF-1: insulin-like growth factor 1; IL-1/6/8: interleukin 1/6/8; PARP: poly-(ADP-ribose)-polymerase; TNFα: tumor necrosis factor-α; VEGF: vascular endothelial growth factor.

Activation of glucose metabolism via the polyol pathway also increases the concentration of diacylglycerol (DAG). Under hyperglycemic conditions, DAG may be formed de novo from intermediate products of glycolytic metabolism. A high concentration of diacylglycerol activates retinal cellular protein kinase C (PKC). On the one hand, DAG can directly activate PKC in the retina, mainly β- and δ-type isozymes, while the activation of PKC-α and -ɛ is also found in the retina of diabetic rats [43]. On the other hand, AGE-mediated signaling pathways, as well as metabolic products of the polyol pathway, are also associated with PKC activation (see Figure 3). Increased PKC activity contributes to the overexpression of matrix proteins and vasoactive mediators. This causes adverse retinal vascular changes, involving structural changes, such as pericyte apoptosis and basement membrane thickening, as well as functional changes, such as increased retinal vascular permeability and retinal blood flow [44,45]. The degeneration of pericyte cells starts with progressive stimulation of PKC-δ signaling, caused by hyperglycemia. The signaling surge stimulates the expression of protein kinase C-δ (PKC-δ, encoded by *Prkcd*) and p38 mitogen-activated protein kinase. This causes dephosphorylation of platelet-derived growth factor receptors (PDGFRs) and decreases their downstream signaling, which in turn results in the self-mediated death of pericytal cells [46]. In addition, activation of PKC may cause overexpression of endothelin-1 (ET-1), which is a potent vasoconstrictor apparently involved in the pathogenesis of diabetic retinopathy (DR), and this can also explain the decreased retinal blood flow [47].

#### 1.3.4. Growth Factors, Cytokines, and Angiogenic Factors

Diabetes leads to reduced cellular proliferation [36] and endothelial cell dysfunction, thereby causing defective angiogenesis [35]. Increased activity of growth factors is an important mechanism in the development of diabetic retinopathy.

Several pro-angiogenic cytokines—including vascular endothelial growth factor (VEGF), insulin-like growth factor I (IGF-1), hepatocyte growth factor (HGF), basic fibroblast growth factor (b-FGF), platelet-derived growth factor (PDGF), pro-inflammatory cytokines, such as IL-1β [48], Il-6 [49], IL-8, tumor necrosis factor-α (TNFα) [50,51], and angiopoietins [52]—are believed to be involved in the pathogenesis of PDR. Endothelial damage due to pericyte loss causes hypoperfusion, which leads to abnormalities in the structure and function of retinal blood vessels. These changes lead to sight-threatening points, which are major complications of DR. The damage to pericytes is associated with endothelial cell damage, along with cotton wool patches, microaneurysms, and dot and blot hemorrhages. Pericyte loss and damage to the endothelium lead to the occlusion of capillaries and local ischemia, which activates hypoxia-inducible factor 1 (HIF-1). Activation of HIF-1 further increases the expression of VEGF, which, along with the other angiogenic factors, i.e., Ang-1 and Ang-2, enhances vascular permeability [53]. In the final stage of the DR pathomechanism, hyperglycemia induces neovascularization and neurodegeneration processes [6]. Neurodegeneration is a result of high-glucose-level-induced down-regulation of several vital neurotrophic and neuroprotective factors, including nerve growth factor (NGF) [54], pigment epithelium-derived factor (PEDF) [55], interphotoreceptor retinoid-binding protein (IRBP) [56], somatostatin (SST), and glucagon-like peptide 1 (GLP-1) [57], whereas neovascularization involves the up-regulation of pro-angiogenic factors, such as VEGF, Ang-1, Ang-2, HGF, and PDGF.

#### 1.3.5. Oxidative Stress

The retina displays high levels of ROS [58,59]. Phototransduction, phagocytosis of photoreceptor outer segments by retinal pigment epithelial (RPE) cells, and oxidization of polyunsaturated fatty acids (PUFAs) in the retina lead to overproduction of ROS [60]. Photoreceptor membranes, containing polyunsaturated fatty acids susceptible to photo-oxidation, are found in the retina at high oxygen tension and under chronic light exposure. Ocular exposure to sunlight and UV light directed at the lens and retina of the human eye can induce retinal degeneration. In photo-oxidation reactions, the chromophore in the eye absorbs light and produces free radicals, such as singlet oxygen, superoxide, and hydroxyl radical. They cause lipid peroxidation, damaging the integrity of biological membranes, which results in ocular tissue damage.

Oxygen-consuming mitochondria in the photoreceptor inner segments also play a major role in retinal ROS production. The retinal hypoxic and hyperglycemic conditions contribute to overexpression of NADPH oxidase (Nox), which leads to overproduction of free radicals, superoxides, and hydrogen peroxide by taking electrons from NADPH and transporting them to molecular oxygen. The Nox system is involved in many processes leading to the overproduction of ROS. For example, the high blood glucose level increases the formation of AGEs and the binding of glycation end products to their receptor (RAGE), which results in increased production of intracellular free radicals via NADPH oxidase [61]. Moreover, activation of protein kinase C via diacylglycerol formation results in subsequent ROS production also via Nox [62].

Research has shown that the Nox system is an important source of oxidative stress in the vascular system. It has been shown that increased Nox 2 activity is associated with ROS overproduction and overexpression of intercellular adhesion molecule-1 (ICAM-1) and VEGF [63].

In contrast, Brownlee and co-authors observed that mitochondrial-derived ROS causes strand breaks in DNA, which in turn activates poly-(ADP-ribose)-polymerase (PARP). High activation of the PARP enzyme inhibits glyceraldehyde phosphate dehydrogenase (GAPDH) activity, which intensifies the accumulation of glycolytic metabolites. The metabolites then activate the AGEs, PKC, polyol, and hexosamine pathways. The authors have proposed that many biochemical and pathological retinal abnormalities induced by diabetes are associated with oxidative stress [64].

It is suggested that the metabolic memory phenomenon and mitochondrial DNA (mtDNA) damage by ROS might be potentially responsible for a prolonged progressive course of DR. As a consequence, mtDNA damage generates sub-normal complexes I and III with reduced membrane potential. This results in the positive feedback loop, where hyperglycemia induces superoxide, which damages mtDNA. This impedes the electron transport chain and results in superoxide overexpression [65].

Research has shown that the damage to mitochondrial DNA (mtDNA) is not uniform, but specific regions are more vulnerable than others. In particular, the displacement loop region (D-loop) of mtDNA, which is responsible for replication and transcription, is exposed to more extensive damage in the course of diabetic retinopathy [66,67]. Mitochondrial DNA methyltransferase (DNMT) is also highly expressed in retinal cells under high glucose conditions, which can highly methylate the D-loop region of mtDNA and cause mitochondrial damage in retinal cells [68]. Moreover, the mtDNA replication/repair system is compromised in DR, leading to decreased gene transcripts and mitochondrial accumulation of enzymes, such as DNA polymerase gamma 1 (POLG1), DNA polymerase gamma 2 (POLG2), and mtDNA helicase. This impairment further hampers the replication/repair process and contributes to dysfunctional mitochondria. The copy numbers of retinal mitochondria are also decreased in diabetic retinopathy [65,69,70]. D-loop integrity and mtDNA replication play important roles in the pathogenesis of diabetic retinopathy, and the high glucose level preferentially damages the D-loop region and the replication machinery in retinal endothelial cells. Additionally, increased reactive oxygen species impair mitochondrial function, damage mtDNA, and accelerate the apoptosis of retinal capillary endothelial cells, pericytes, and neurons [71,72]. Overexpression of mitochondrial superoxide dismutase (MnSOD) protects against the damage of retinal mtDNA and prevents the development of diabetic retinopathy [70].

In addition, phototoxic reactions can modify histidine, tryptophan, and cysteine, some amino acids of the lens, leading to changes in their physical properties [73].

During normal homeostasis, retinal cells maintain a balance between pro- and anti-oxidative processes. However, prolonged hyperglycemia in DM increases hypoxia and advanced glycation ROS production. It also accelerates several intracellular alterations, such as oxidative stress, mitochondrial dysfunction, and it causes endoplasmatic reticulum stress, which are all responsible for autophagy flux impairment and apoptosis [58]. Overproduction of ROS disrupts redox homeostasis, which in turn results in neuronal cell loss, vascular abnormalities, and the occurrence of advanced DR [74].

Hypoxia and hyperglycemia, abnormal polyol and hexosamine pathways, increased diacylglycerol level, non-enzymatic glycation of proteins with their end products, and protein kinase C activation lead to a series of pathological events, including overproduction of ROS, oxidative stress, activation of pro-angiogenic factors, and finally, inflammation with leukostasis.

#### 1.3.6. Inflammation with Leukostasis

Diabetic retinopathy is a disease of the retinal neurovascular unit, which refers to the functional coupling and interdependency of neurons, glia, and vasculature. Inflammatory responses play a prominent and complex role in further propagating the pathways in diabetic retinopathy through cytokines, adhesion molecules, VEGF signaling, enhanced RAGE expression, changes in nitric oxide regulation, and NF-κB signaling [75]. Leukocytes are characterized by large cell volumes, high cytoplasmic rigidity, and a natural tendency to adhere to the vascular endothelium via cellular adhesion molecules. This attachment, under hyperglycemic conditions, could result in non-perfusion and, ultimately, proliferative diabetic retinopathy. Moreover, inflammation in the retina leads to increased intraocular blood pressure, formation of new weak vessels, and increased permeability, resulting in hemorrhages in the retina and leukostasis. Leukostasis is manifested with the clustering of neutrophils in microcirculation. This is a significant factor in diabetic retinopathy, which is a progressive microvascular disease. Moreover, leukostasis is an early inflammatory response in DR, leading to damaging effects on retinal circulating leukocytes and vascular endothelium [76].

The increase in blood glucose/dyslipidemia parallels the increased leukocyte trapping/leukostasis. It is associated with pathologically reduced capillary density and acellular capillaries in the later stages of diabetic retinopathy. Hyperglycemia activates circulating leukocytes, which are then prone to adhere to vascular walls. This leads to a progression from occasional transient leukocyte trapping to multiple sites of prolonged leukostasis with fewer successful reperfusions until networks of capillaries are permanently occluded in DR [77].

Kaji Y. and co-authors [78] reported that diabetic non-transgenic mice exhibit a three-fold increase in the number of adherent leukocytes compared to non-diabetic control mice. Similarly, leukostasis increases in diabetic retinopathy. The number of adherent leukocytes is two-fold higher in diabetic RAGE transgenic mice compared to non-diabetic mice, even under normoglycemic conditions. However, a systemic application of RAGE significantly reduces the number of adherent leukocytes in the retinas of diabetic non-transgenic and diabetic RAGE transgenic mice. Adherent leukocytes are detected on the arterioles, venules, and capillaries in the retinal vasculature.

Inflammatory cytokines, chemokines, and growth factors, such as TNF-α, IL-1β, hepatocyte growth factor, insulin-like growth factor-1, IL-6, MCP-1, and histamine, promote leukostasis in DR. Moreover, the increased expressions of inflammatory factors, including cell adhesion molecules, such as GMP-140, ICAM-1, CD11/CD18, and VCAM-1, are involved in inflammation processes with leukostasis. Leukocytes migrate through the endothelium into the extravascular space, where they differentiate into activated macrophages and secrete various cytokines and inflammation-related factors. Antibodies against CD-18 or ICAM-1, or genetic knock-out of these genes in animals, can inhibit leukostasis and decrease BRB breakdown [76]. Neutrophils of diabetic animals exhibit higher levels of surface integrin expression, such as CD18, and integrin-mediated adhesion. These mediators disrupt the cell–cell junctions, resulting in BRB breakdown. Chronic hyperglycemia increases the expression of chemokines, including CCL2, which increase leukostasis, diapedesis, and influx of monocytes into the retina and extravascular space [76].

Studies have also shown that leukostasis is a parameter, which could help predict the severity of retinal vascular lesions in proliferative DR [79].

DR can be viewed as a disease of the retinal neurovascular unit, which damages the functional coupling and interdependency of neurons, glia, and vasculature. Inflammatory responses play a prominent and complex role in further propagating the pathways in DR through cytokines, adhesion molecules, VEGF signaling, enhanced RAGE expression, changes in nitric oxide regulation, and NF-κB signaling. Hyperglycemia, oxidative stress, formation of advanced glycation end product, and hypertension all contribute to inflammation in DR. Leukostasis, i.e., the attachment of leukocytes to the luminal endothelium of capillaries, is a prominent cellular event in DR. The increased expression of inflammatory factors, including cell adhesion molecules, such as GMP-140, ICAM-1, CD11/CD18, and VCAM-1, promotes leukostasis. The dysregulation of cellular interactions in the processes of neurovascular coupling leads to DR even before the observable vascular lesions appear.

#### 1.3.7. Non-Coding RNAs as Biomarkers in DR

Recently, non-coding RNAs (ncRNAs), including microRNAs (miRNAs), lncRNAs, and circRNAs, have appeared to play the role of specific biomarkers for the early diagnosis and monitoring of DR, as well as promising therapeutic agents for DR treatment [80]. ncRNAs can be secreted into the body fluid to play a role in the pathomechanism of DR, and their primary pathway is executed in the exosomes.

miRNAs are RNA molecules belonging to the family of small non-coding RNAs 20–25 nucleotides long, located within the introns and exons of protein-coding genes or in the intergenic regions. miRNAs are involved in gene silencing by repressing protein synthesis through imperfect binding to the three prime untranslated region (3′-UTR) of the target mRNA, leading to mRNA degradation. MicroRNAs play a key role in the regulation of gene expression, and they are also involved in many important biological processes, such as cell proliferation, differentiation, angiogenesis, growth control, organogenesis, and apoptosis [81]. Moreover, they are also involved in the occurrence and development of various diseases, including DR.

Cells release miRNAs into the bloodstream, where they remain viable for approximately 2 weeks. Their stability in plasma/serum/urine upon freeze–thawing, efficient recovery, and the availability of quantitative detection methods make them a specific biomarker. Furthermore, the use of circulating miRNAs as biomarkers allows for non-invasive testing, which reduces patient discomfort and may increase patient compliance. Studies conducted in recent years have shown dysregulation of microRNAs in DR. A discovery of new specific microRNAs as prognostic biomarkers for DR facilitates early detection of the disease and allows us to constantly monitor its progression. Understanding how miRNAs coordinate disease progression may allow us to better understand the disease itself and initiate new therapeutic approaches.

Some exosomal miRNAs are involved in the regulation of angiogenesis, neovascularization, and apoptosis, as well as inflammatory processes, neurodegenerative processes, and oxidative stress, which play a role in the pathophysiology of DR. Recent reviews indicate that dysregulation of miRNAs is associated with the development of DR, especially in retinal cell dysfunction. For instance, five miRNAs (hsa-miR-195-5p, hsa-miR-20a-5p, hsa-miR-20b-5p, hsa-miR-27b-3p, and hsa-miR-451a) have been validated as biomarkers for stratification of DR stages [82], while miRNAs such as let-7a-5p and miR-211 have been shown to be up-regulated in DR and associated with increased proliferation of retinal microvascular endothelial cells and suppression of expression of downstream target gene *SIRT1*, respectively [83]. Additionally, it has been observed that the miR-21 level is increased in DR and potentially induces angiogenesis via targeting the phosphatase and tensin homolog (PTEN), leading to activation of AKT and ERK1/2 signaling pathways and thereby enhancing HIF-1α and VEGF expression [84]. On the other hand, the down-regulation of miR-126 in T2DM-DR compared to T2DM-noDR may be related to endothelial damage, as it protects vascular endothelial cells and also facilitates vascular endothelial growth factor- A (VEGF-A) signaling [85].

Zampetaki et al. [86] conducted extensive research, which represents an important advance in the field of miRNA biomarkers for DR. The authors performed a comprehensive analysis of miRNA expression profiles in DR using samples from two DR-related clinical trials in patients with T1DM: PROTECT-1 with non-proliferative retinopathy and PREVENT-1 without retinopathy. They identified 29 miRNAs, which were differentially expressed among DR patients and controls. They included miR-27b-3p and miR-320a-3p, which were significantly associated with a high risk of DR. Furthermore, the authors used cultured human endothelial cells to investigate the potential mechanism underlying these miRNA effects and identified thrombospondin-1 as a common target of miR-27b-3p and miR-320a-3p. These findings suggest that the above miRNAs may play a role in the development and progression of DR. However, it is important to note that the study used a candidate miRNA approach, which may not be suitable for identifying the best signature for DR due to the ability of a single miRNA to target multiple gene transcripts. Nonetheless, this study provides valuable insights into dysregulated miRNAs in DR and highlights the need for further research to validate these findings and explore the potential use of miRNAs as therapeutic targets or agents in the treatment of retinopathy in diabetics.

Santovito D. et al. [87] found higher expressions of miR-25-3p and miR-320b (another member of the miR-320 family), whereas changes in miR-320a-3p appeared to be statistically insignificant, as in Zampetaki’s [86] study. Authors pointed out that the miRNAs identified in their experiment are enriched in other cell types, such as fibroblasts, stem cells, and leukocytes, which might make them different.

Additionally, miR-23a, miR-320a, and miR-320b had increased expressions in vitreous humors in PDR patients compared to patients with macular hole (MH) [88], while miR-204 and let-7c were significantly down-regulated in PDR patients in comparison to MH patients [89].

In contrast, specific miRNAs, such as miR-21, miR-181c, and miR-1179 [90], miR-93 [91], miR-221 [27], are potential biomarkers for diabetic retinopathy, which enable diagnosis, prognosticate DR, and also monitor its progression. Moreover, the study revealed that miRNA-181c might be associated with vascular proliferation in high glucose (HG) [90].

In addition, miRNAs appeared to be important regulatory molecules in the pathogenesis of DR. A study by Wang et al. [92] showed that miR-409-5p might play a key role in neovascularization in DR. The authors investigated the expression of miR-409-5p in diabetic retinal tissues, mouse retinal microvascular endothelial cells, and vitreous fluid of proliferative DR patients. The experiment explored the effect of miR-409-5p on retinal neovascularization in vitro and in vivo and aimed to provide a novel insight into the regulation pattern of miR-409-5p in DR. Knock-down of miR-409-5p suppresses VEGF-induced retinal neovascularization in vitro, while overexpression of miR-409-5p promotes proliferation, migration, tube formation and increases VEGF expression and secretion. PPARα is a downstream target of miR-409-5p, and its overexpression negates the promotion of miR-409-5p overexpression in the proliferation, migration, and tube formation of retinal microvascular endothelial cells (mRMECs). Knock-down of miR-409-5p attenuates retinal neovascularization in vivo and reduces the number of acellular capillaries per mm2 and the levels of neovasculogenic factors, including VEGF, TNF-α, ICAM-1, and MCP-1, in diabetic retinal tissues. The expression of miR-409-5p in the vitreous fluid of proliferative DR patients was higher than that of control patients, and miR-409-5p was markedly increased in the retinal tissues of STZ-induced DM mice and db/db mice in comparison with control mice. These findings suggest that miR-409-5p may be a key miRNA associated with neovascularization in DR and that anti-miR-409-5p therapy may represent a novel therapeutic strategy for DR in the future.

Additionally, the relationship between miRNA expression and oxidative stress signaling pathways in DR is an interesting topic of active research.

miRNA has been shown to regulate the expression of many gene transcripts in diabetic retinopathy, which implicates its role in the antioxidant activity of plant extracts and flavonol compounds. For instance, high glucose and vitamin D treatment in human retinal endothelial cells can reduce miR-93 transcript, ROS production, MDA content, and elevated GSH activity [93]. miR-93 has also been shown to target SIRT1 in rats with streptozotocin-induced diabetes, thereby contributing to changes indicative of oxidative stress and inflammation, which can be reversed by SIRT1 overexpression [94]. Another miRNA, miR-338-3p, negatively regulates glutamine transporter SLC1A5 expression, leading to ferroptosis [95]. In addition, repression of mitochondrial SIRT transcripts SIRT3, SIRT4, and SIRT5 by miR-1, miR-19a, and miR-320a, respectively, is implicated in oxidative damage in diabetic retinopathy [96].

Notably, miRNA-145 overexpression can reduce ROS and malondialdehyde (MDA) levels, increase superoxide dismutase activity, and decrease hyperglycemia-induced oxidative stress and retinal endothelial cell apoptosis [97]. Moreover, miR-183 activates the PI3K/Akt/VEGF signaling pathway and increases CD34, eNOS, and ROS [98]. On the other hand, miR-27 inhibits the Nox 2 signaling pathway by downregulating P13K/AKT/mTOR, thus reducing ROS production [99].

**Figure 4:**
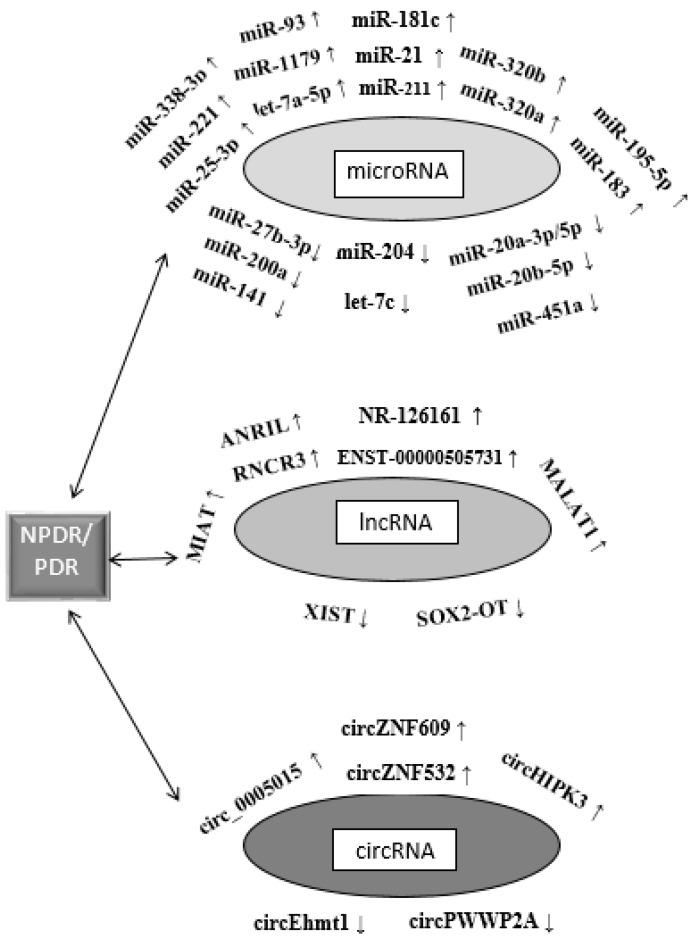
The main non-coding RNAs associated with the pathomechanism of DR.

Activation of the polyol pathway by hyperglycemia increases the activity of aldose reductase, which in turn decreases the expressions of miR-200a and miR-141 (these miRNAs are regulators of Kelch-like ECH-associated protein 1 (KEAP-1)), suppressing nuclear factor erythroid 2-related factor 2 (NRF2) and resulting in increased ROS and oxidative stress. However, aldose reductase deficiency in the renal cortex up-regulates miR-200 and miR-141, releases the KEAP-1 suppression of NRF2, and ameliorates oxidative stress, preventing kidney fibrosis [100]. The main ncRNAs associated with the pathomechanism of DR are shown in Figure 4.

The second increasingly studied group of RNA molecules belonging to the family of non-coding RNAs are long non-coding RNAs (lncRNAs). lncRNAs are RNA molecules, which do not have significant protein-coding potential and are over 200 nucleotides long, making them a distinct class of transcripts. More than 100,000 lncRNA genes have been identified in humans. They can be involved in multiple biological processes through different molecular mechanisms and play a vital role in various biological processes, such as post-transcriptional gene regulation and epigenetic gene silencing [101]. lncRNAs have versatile and critical roles in various diseases, such as cancers [102], neurodegenerative diseases [103], diabetes, and complications such as diabetic retinopathy [104].

lncRNAs can regulate gene expression in both cis and trans regulatory relationships and form regulatory networks with coding mRNAs. lncRNAs can be classified as nuclear-retained or cytoplasmic. The introns present in both protein-coding and non-protein-coding genes are the major source of regulatory non-coding RNAs, including lncRNAs [101]. Nuclear-retained lncRNAs play important roles in transcriptional regulation, while one of the key roles of cytoplasmic lncRNAs in gene expression regulation is played through miRNA sponge activity, where lncRNAs can act as competing endogenous RNAs (ceRNAs), regulating mRNA expression by sequestering miRNAs [105]. Furthermore, lncRNAs have unique expression patterns and were most often found to be up-regulated in different contexts related to DR.

The X inactive specific transcript (XIST) is a widely studied lncRNA. It has been found to be down-regulated in HG-treated Müller retinal cells isolated from a diabetic mouse model and human Müller retinal cell line and ARPE-19. XIST has been found to exert its functions by sponging miRNAs, and its overexpression produces miR-21-5p down-regulation, which could determine the modulation of VEGF signaling. Overexpression of XIST has a protective effect on apoptosis and migration in ARPE-19 treated under HG conditions, also reducing the production of pro-inflammatory cytokines in HG-treated mice and human Müller cells [106,107].

Myocardial infarction associated transcript (MIAT) is a lncRNA, whose potential protective role in DR has been identified. MIAT is an intergenic lncRNA, which is mostly conserved through species and likely forms a part of the nuclear matrix. MIAT is located on the 22q12.1 locus associated with myocardial infarction susceptibility [108]. MIAT plays an important role in various human biological processes, such as neuronal survival and the formation of nuclear bodies [109,110]. Li et al. [111] demonstrated that plasma samples from patients with DR showed overexpression of MIAT compared to both patients without DR and the control group. Moreover, samples from animal diabetic retinas and endothelial cells cultured in a high glucose medium showed up-regulation of MIAT and involvement in regulation of the endothelial cell function and pathological angiogenesis [112].

Another lncRNA, i.e., retinal non-coding RNA 3 (RNCR3), has also been found to play a role in protecting against DR. This lncRNA has been linked to neuronal and oligodendrocyte differentiation and atherosclerosis-related vascular dysfunction [113]. Knock-down of RNCR3 has been shown to alleviate retinal microvascular leakage and inhibit migration and tube formation by RF/6A cells under HG stress. RNCR3 is significantly up-regulated under HG stress in RF/6A cells and retinas of diabetic mice, as well as in fibrovascular membranes of PDR patients. Inhibition of RNCR3 may therefore be a potential treatment option for preventing DR-related retinal abnormalities [114]. Moreover, another study demonstrated that RNCR3 knock-down significantly inhibits retinal reactive gliosis [115]. Inhibition of RNCR3 leads to a significant reduction in the release of cytokines: IL-2, IL3, IL-4, IL-5, IL-9, IL-13, IL-17, MCP-1, VEGF, and TNF-α. Moreover, administration of intravitreal RNCR3 shRNA impedes glial cell reactivity and induces reductions in cytokines in diabetic mice. Furthermore, shRNA-mediated knock-down of RNCR3 reduces acellular capillaries and retinal vascular leakage in diabetic retinas. There is also a significant decrease in cytokines, such as MCP-1, TNF-α, and VEGF-A, in the retinas of diabetic mice following intravitreal RNCR3 shRNA administration. The experiment showed that RNCR3 knocks down the reduction in apoptotic retinal cells, prevents HG-induced retinal neurodegeneration, and improves visual function. Overall, these findings suggest that lncRNAs such as RNCR3 play a crucial role in the protective mechanisms against DR and provide a potential therapeutic target for DR treatment [115].

Metastasis-associated lung adenocarcinoma transcript 1 (MALAT1) is another lncRNA widely studied in DR. Studies have shown that dysregulation of MALAT1 expression plays a key role in the pathogenesis of diabetic retinopathy. Hypoxia—which is a central pathophysiological phenomenon in diabetes-targeted organs—induces transactivation of the MALAT1 promoter through enhanced activity of the hypoxia-inducible factor-1α (HIF-1α) in vitro [116]. In the retina, MALAT1 can modulate the progression of neurodegeneration by activating the cyclic adenosine monophosphate (cAMP)—a response element binding protein (CREB) and p38 MAPK signaling pathway. MALAT1 overexpression can dysregulate microvascular growth, even disturbing the function of retinal endothelial cells, while MALAT1 knock-down ameliorates DR in vivo and regulates endothelial cell functions (cell proliferation, migration, and tube formation) in vitro via cross-talk between MALAT1 and the p38 MAPK pathway [117].

Puthanveetil et al. [118] documented that MALAT1 knock-down in human umbilical vein endothelial cells (HUVECs) under hyperglycemic conditions downregulates serum amyloid antigen 3 (SAA3) activation, subsequently reducing the RNA and protein expressions of key inflammatory mediators (IL-6 and TNF-α) implicated in diabetic complications. Biswas et al. [119] made a particularly interesting observation by revealing that MALAT1 can impact the expressions of inflammatory transcripts through its association with components of the Polycomb repressive complex 2 (PRC2) in diabetes. PRC2 is a chromatin-modifying enzyme, which catalyzes the methylation of histone H3 at lysine 27. Moreover, MALAT1, TNF-α, and IL-6 expressions are upregulated in the vitreous humors of diabetic patients. Additionally, Radhakrishnan et al. [120] observed that siRNA-mediated knock-down of MALAT1 in human endothelial cells of the retina (HRECs) modulates antioxidant defense in DR. MALAT1 down-regulation leads to suppression of inhibitor Keap1 and dissociation of NRF2, thus stimulating the transcription of antioxidant and detoxifying genes.

The anti-sense non-coding RNA in the INK4 locus (ANRIL) expression dysregulation has also been identified as a key mechanism in the pathogenesis of diabetic retinopathy. ANRIL is known to regulate VEGF expression and function in DR. Thomas et al. [121] observed that ANRIL-knock-out diabetic mice and ANRIL-silenced human retinal endothelial cells (HRECs) with high glucose levels display reduced levels of VEGF expression via binding p300—the enhancer of Zeste homolog 2 (EZH2) of the PRC2 complex—and miR-200b. Knocking down the ANRIL levels using siRNAs prevents VEGF up-regulation at the mRNA and protein levels, thereby inhibiting cell proliferation and tube formation. Furthermore, Toraih et al. [122] observed that the plasma of T2DM with DR displays up-regulation of ANRIL compared to both patients without DR and healthy subjects. Another study described no differences in ANRIL expression in the serum of DM patients compared to healthy subjects, whereas patients with NPDR and PDR—unlike both DM patients without DR and healthy individuals—demonstrated up-regulation in serum, aqueous humor, and vitreous humor [123].

In contrast, the low level of SOX2 overlapping transcript (SOX2OT) is linked to neurodegenerative complications. In humans, SOX2OT is highly expressed in the brain and may be involved in the regulation of retinal neural function, thereby affecting the retinal neurodegeneration process [124]. Decreased expression of SOX2OT is reported in the retinas of STZ-induced diabetic mice as well as retinal ganglion cells (RGCs) in the presence of high glucose or oxidative stress and regulates NRF2/HO-1 signaling activity, thereby proving its antioxidant function and neuroprotective role in diabetes-related retinal neurodegeneration in vivo. These findings suggest that SOX2OT knock-down may be a potential treatment option for diabetes-induced retinal [125]. Additionally, mice with diabetic nephropathy compared to control mice displayed decreased levels of SOX2-OT, and this result was confirmed in cultured human podocytes and mesangial cells [126].

Recent research has shown that lncRNAs have significant potential as disease-specific biomarkers for the diagnosis of DR. In particular, two up-regulated lncRNAs have demonstrated high sensitivity in distinguishing PDR patients from NPDR patients and the control group. These lncRNAs exhibit a disease-state-specific pattern, making them good candidates for biomarker development. The high sensitivity and specificity of these lncRNAs in distinguishing PDR patients suggest that they could serve as potential biomarkers in the prediction and diagnosis of DR. In particular, blood ENST00000505731 and NR-126161 lncRNAs have shown a potential diagnostic value for DR. However, the authors explained that the study was limited by its sample size, and future prospective screening studies with larger groups are necessary in order to increase the sensitivity of the assay and determine whether certain sub-stages of DR can be detected through lncRNA expression levels and/or a combination of lncRNA phenotypes before clinical diagnosis. Overall, this study provides valuable insight into the potential use of lncRNAs as diagnostic biomarkers for DR, which could have implications for improving early detection and treatment of this debilitating disease [127]. Dysregulated lncRNAs involved in diabetic retinopathy are summarized in Table 1.

Circular RNAs (circRNAs) are classified as a sub-group of long non-coding RNAs. Yet, it has recently been reported that some of them may encode proteins [128,129]. They are a distinct type of RNA molecules characterized by a covalent bond linking the 3′ and 5′ ends, which results in a closed-loop structure. Unlike linear RNA, circRNAs are formed by a non-canonical event called back-splicing, where the 3′ end of an exon is covalently linked to the 5′ end of another exon [130]. They are highly stable due to their circular structure, which resists degradation by RNase R—an exonuclease, which degrades almost all linear forms of RNA. They are characterized by specific expression and complex regulation, with tissue-specific or developmentally specific expression patterns. They regulate gene expression by acting as miRNA sponges. Some of them are believed to regulate the function of microRNAs (miRNAs) and play a role in transcriptional control. Moreover, circRNAs can bind to RNA-binding proteins (RBPs) and are involved in the regulation of translation [131]. Moreover, circRNAs can code small proteins and peptides. In comparison to their linear host gene products, circRNA-encoded proteins or peptides perform independent biological functions. For example, circAKT3, which encodes a 174 amino acid novel protein, can negatively regulate the receptor tyrosine kinase/phosphatidylinositol 3′-kinase (RTK/PI3K) pathway [132].

Most circular transcripts are tissue-specific. circRNA accumulation is higher in slowly dividing cells, e.g., in the brain, compared to rapidly dividing cells, e.g., in the liver [133]. High accumulation of circRNA is also found in peripheral blood (especially in erythrocytes and platelets), in healthy organs, cancerous tumors, and cell lines, as well as in body fluids, such as urine and saliva. Large amounts of circRNA have been detected in exosomes [134,135].

The fact that circRNAs are characterized by high stability and specificity depending on the type of cells or various pathological conditions makes them good potential biomarkers. Therefore, circRNAs have become a new research hotspot in the field of RNA, with thousands of human circRNAs being identified using molecular biology and bioinformatics methods.

Zhang SJ et al. [136] found that one of the circular RNAs derived from the HAS2 gene locus—circ_0005015—was significantly up-regulated in diabetic retinas, vitreous samples, plasma fractions of whole blood, and preretinal fibrovascular membranes (FVMs) of DR patients. Functional assays have revealed that circ_0005015 can regulate the retinal endothelial cell function by acting as a miRNA sponge, and it has been proven to play a vital role in the progression of DR by regulating the growth, proliferation, migration, and tube formation of retinal vascular endothelial cells. circRNA homeodomain-interacting protein kinase 3 (circHIPK3) and circRNA zinc finger protein (cZNF609) also play a similar role [137,138]. circRNA zinc finger protein 532 (circZNF532), produced in the cytoplasm of pericytes, is another such molecule. CircZNF532 regulates the expression of NG2, LOXL2, and CDK2 proteins, which are essential for maintaining vascular stability. Notably, overexpression of circZNF532 reduces the diabetic effect on microangiopathy, protecting against diabetes-induced retinal pericyte degeneration and vascular dysfunction, highlighting its significance in DR pathogenesis. Moreover, circZNF532 acts as a miR-29a-3p sponge to regulate gene expression, making it a potential therapeutic target in DR treatment [139]. In contrast, Wang et al. [140] revealed that circZNF532 contributed to expedited high-glucose-concentration-induced human retinal microvascular endothelial cell (hRMEC) dysfunction via sponging miR-1243 and activating arginine methyltransferase 1 (CARM1).

Hypoxia-induced circRNA euchromatic histone lysine methyltransferase 1 (circEhmt1) in the nucleus of pericytes regulates the pericyte–endotheliocyte cross-talk, which plays an important role in the pathogenesis of DR [141]. Another circRNA PWWP domain, containing 2A (circPWWP2A), acts as a miR-579 sponge and up-regulates the angiopoietin-1/Occluding/Sirtuin 1 proteins, thus contributing to the promotion of DR progression [28]. Dysregulated circRNAs involved in DR are summarized in Table 2.

Data in the literature show that the role of miRNAs, lncRNAs, and circRNAs is not fully understood, and their participation in regulatory mechanisms has to be investigated. Researchers mainly use endothelial cells, glial, or RPE cells as biological material. Visual cells, bipolar cells, horizontal cells, amacrine cells, and reticular cells are more difficult to obtain the research material for [142]. Moreover, it is not easy to apply the ncRNAs detected in experimental studies to clinical practice. However, the use of ncRNAs as diagnostic biomarkers in common clinical practice still requires many studies. An independent expression analysis should be performed on a large sample of patients, taking into account factors such as age, ethnicity, lifestyle, and overall health. Further research is necessary on the mechanisms of ncRNA secretion into the extracellular environment and their direct and indirect effects on distant cells taking up these molecules from circulation. It is also necessary to improve and standardize the method for the isolation of these RNAs from biological material, quantification, analysis, and normalization of the data. In the future, research on this new class of biomarkers may be included in the early and precise diagnosis of diabetes and its complications, including DR, and will also help select the appropriate therapy for a specific patient. However, further research on the practical use of ncRNAs will enable clinicians to diagnose DR earlier and make the treatment effective [142].

The correlation of ncRNA profiles with DR indicates their involvement in molecular pathways, which are important in the pathomechanism of diabetic retinopathy. ncRNAs are involved in the regulation of retinal cell damage, vascular changes in choroidal vessels, inflammation in RPE cells, leukostasis, neovascularization, neurodegeneration, and antioxidant defense capacity [142]. Therefore, ncRNA profiling in body fluids has increasingly promising applications in the prognosis, monitoring, and diagnosis of DR. Clinically useful molecular diagnostic tests are one of the most important applications of ncRNA profiling. ncRNAs may also be more effective as targeted drugs than traditional therapeutic drugs. The development of therapeutic strategies targeting excessive cell proliferation and apoptosis, inflammatory responses, and oxidative stress, as well as analysis of an ncRNA panel, may significantly impact the treatment of DR.

## 2. Conclusions

The exact mechanism responsible for the development and progression of DR is not fully understood, but extensive research has revealed several key molecular processes involved in this disease. The formation of ROS due to elevated glucose leads to the loss of neuronal and pericyte cells, blocked capillaries, and distortion of the microvascular structure of the retina. Furthermore, the degeneration of pericyte cells is caused by progressive stimulation of protein kinase C-delta (PKC-δ) signaling. The breakdown of the blood–retina barrier (BRB) is a crucial event in the pathophysiology of DR, leading to vascular leakage and edema in retinal tissues. Oxidative stress also contributes to early clinical hallmarks of diabetic retinopathy, including a thickened basement membrane, pericyte apoptosis, and mitochondrial dysfunction, which further exacerbate BRB impairment. These molecular processes and pathways interplay in a complex manner, resulting in the occurrence of characteristic clinical features of diabetic retinopathy, such as elevated vascular flow, vascular lesions, inflammation, neovascularization, and neurodegeneration. Understanding these mechanisms is essential for designing effective treatment modalities, which can prevent or slow down the progression of diabetic retinopathy, as it is a substantial burden for the affected individuals and socio-economy. Furthermore, studies have shown that diabetes can influence circulating non-coding RNA expression, with some of it serving as a potential biomarker for DR diagnosis. However, more research is needed to properly investigate the mechanisms underlying the involvement of non-coding RNAs (miRNAs, lncRNAs, circRNAs) in DR and identify a non-invasive diagnostic and prognostic ncRNA signature of DR, as well as to explore the distinct pathways and ncRNAs, which underlie the development of DR in type 1 or type 2 diabetes. This will also allow the development of targeted therapies with fewer side effects for this sight-threatening disease.

## Figures and Tables

**Table 1:** Dysregulated lncRNAs involved in diabetic retinopathy.

lncRNAs	Sample	Dysregulation	Pathogenic Effects	Reference
**XIST**	Human retinal pigment epithelial ARPE-19 cells	Down-regulated	Apoptosis, migration	[106]
	Mouse retinal Müller cells (mMCs), human retinal Müller cell line (HMCs)	Down-regulated	Inflammation	[107]
**MIAT**	Human plasma, ARPE-19 cells	Up-regulated	Decreases retinal pigment epithelial cells’ viability	[111]
	HMVECs, RF/6A, RPE, RGC, and Müller cells	Up-regulated	Angiogenesis, proliferation, migration, and survival of endothelial cells	[112]
**RNCR3**	Retinas of DM mice, RF/6A cells	Up-regulated	Retinal vascular functions, proliferation, migration, pro-inflammatory activation of ECs and VSMCs	[113]
	Retinas of DM mice, Müller cells	Up-regulated	Retinal vascular functions, release of several cytokines	[115]
**MALAT1**	Retinas of DM mice, Müller cells, primary retinal ganglion cells (RGCs)	Up-regulated	Retinal neurodegeneration	[117]
	Human umbilical vein endothelial cells (HUVECs)	Up-regulated	Inflammation	[118]
	Human retinal endothelial cells (HRECs), diabetic mice, vitreous humors (VH) of DM patients	Up-regulated	Inflammation	[119]
	Human endothelial cells of the retina (HRECs)	Up-regulated	Oxidative stress	[120]
**ANRIL**	HRECs, diabetic mice	Up-regulated	Regulates VEGF, proliferation, migration, tube formation, vascular permeability	[121]
	Blood serum, aqueous fluid, and vitreous fluid: T2DM patients with NPDR and PDR vs. T2DM without DR	Up-regulated	-	[123]
	Blood serum: T2DM patients vs. HC	No difference	Up-regulated VEGF, neovascularization, angiogenesis	[123]
	Blood plasma from T2DM patients with DR/without DR	Up-regulated	No association with DR progression	[122]
**SOX2-OT**	Retinas of DM mice, RGCs	Down-regulated	Retinal neurodegeneration	[125]
**ENST-00000505731** **NR-126161**	Whole blood: T2DM patients with NPDR and PDR vs. HC	Up-regulated	Can serve as diagnostic biomarkers to detect DR early and monitor its progression	[127]

**Table 2:** Dysregulated circRNAs involved in diabetic retinopathy.

circRNAs	Sample	Dysregulation	Pathogenic Effects	Reference
**circ_0005015**	Diabetic retinas, vitreous samples, plasma fractions of whole blood, preretinal fibrovascular membranes (FVMs) of DR patients	Up-regulated	Promoted the growth, proliferation, migration, and tube formation of retinal vascular endothelial cells	[136]
**circHIPK3**	Retinas of DM mice	Up-regulated	Endothelial proliferation, migration, angiogenesis, inflammation, vascular dysfunction	[137]
**circZNF609**	HUVECs, retinas of DM mice, Müller cells	Up-regulated	Migration, tube formation, angiogenesis, apoptosis, oxidative stress	[138]
**circZNF532**	Human retinal pericytes (ACBRI-183) and HRVECs retinas of DM mice, vitreous humors of DM patients	Up-regulated	Pericyte degeneration, cell viability, angiogenesis, breakdown of BRB	[139]
	Blood serum: T2DM patients vs. HC human retinal microvascular endothelial cells (hRMECs)	Up-regulated	Proliferation, migration, angiogenesis, inflammation	[140]
**circEhmt1**	Mice, retinal microvascular pericytes, and endotheliocytes	Down-regulated	Migration, tube formation, angiogenesis, apoptosis, neovascularization, inflammation	[141]
**circPWWP2A**	DM mice, human retinal microvascular endothelial cells (HRVECs), human retinal pericytes	Down-regulated	Retinal microvascular leakage, angiogenesis, apoptosis, neovascularization	[28]
